# Baculovirus as a Tool for Gene Delivery and Gene Therapy

**DOI:** 10.3390/v10090510

**Published:** 2018-09-19

**Authors:** Chikako Ono, Toru Okamoto, Takayuki Abe, Yoshiharu Matsuura

**Affiliations:** 1 Department of Molecular Virology, Research Institute for Microbial Diseases, Osaka University, Osaka 565-0871, Japan; chikaono@biken.osaka-u.ac.jp (C.O.); toru@biken.osaka-u.ac.jp (T.O.); 2 Division of Infectious Disease Control, Center for Infectious Diseases, Kobe University Graduate School of Medicine, Kobe, Hyogo 650-0017, Japan; atakayu@med.kobe-u.ac.jp

**Keywords:** baculovirus expression vectors, insect cells, mammalian cells, gene therapy

## Abstract

Based on its ability to express high levels of protein, baculovirus has been widely used for recombinant protein production in insect cells for more than thirty years with continued technical improvements. In addition, baculovirus has been successfully applied for foreign gene delivery into mammalian cells without any viral replication. However, several CpG motifs are present throughout baculoviral DNA and induce an antiviral response in mammalian cells, resulting in the production of pro-inflammatory cytokines and type I interferon through a Toll-like receptor (TLR)-dependent or -independent signaling pathway, and ultimately limiting the efficiency of transgene expression. On the other hand, by taking advantage of this strong adjuvant activity, recombinant baculoviruses encoding neutralization epitopes can elicit protective immunity in mice. Moreover, immunodeficient cells, such as hepatitis C virus (HCV)- or human immunodeficiency virus (HIV)-infected cells, are more susceptible to baculovirus infection than normal cells and are selectively eliminated by the apoptosis-inducible recombinant baculovirus. Here, we summarize the application of baculovirus as a gene expression vector and the mechanism of the host innate immune response induced by baculovirus in mammalian cells. We also discuss the future prospects of baculovirus vectors.

## 1. Introduction

Baculovirus has been widely used as a gene expression tool for more than thirty years based on its ability to express high levels of proteins in insect cells. Along with the progress of baculovirus research, recombinant baculoviruses have been shown to be capable of entering into various mammalian cells and of expressing foreign genes under the control of mammalian promoters without any viral replication [1,2,3,4,5,6]; as a result, they have been applied for gene delivery into mammalian cells. The advantages of the baculovirus gene expression system are its high transgene capacity; its enabling of both easy construction of recombinant virus with a bacmid system [7] and posttranslational modification with a eukaryotic system such as glycosylation; and, especially in mammalian cells, its replication-defect property—namely, the absence of primary antibody and low cytotoxicity compared to mammalian virus-derived vectors. In addition to achieving an efficient gene delivery, baculovirus has also been shown to stimulate the host antiviral immune responses in mammalian cells [8,9,10,11] and to confer protection from lethal virus infection [8,11] and progressive tumor metastasis in mice [12]. Here, we describe the properties of baculovirus as a tool for gene delivery, gene therapy and vaccine development.

## 2. Baculovirus as a Vector for Gene Delivery

### 2.1. Genome Structure and Very-Late Gene Expression of Baculovirus

Among Baculoviridae, Nucleopolyhedrovirus (NPV) is a large circular double-stranded-DNA (dsDNA) virus with a genome of 80–150 kbp containing over 100 genes [13]. The typical baculovirus expression vector system (BEVS) is based on *Autographa californica* multiple NPV (AcMNPV) or *Bombyx mori* NPV (BmNPV); the former is often used for culture cell-based expression [7,14], and the latter for insect (silkworm)-based expression [15].

Baculovirus gene expression is regulated in a phase-dependent manner, with the immediate-early phase of regulation occurring first, followed by the delayed-early, late and very-late phases [16]. In BEVS for recombinant protein production in insect cells, the promoters of very-late genes, *polyhedrin (polh*) and *p10*, coding a major component of the occlusion body (polyhedra) and polyhedra envelope of NPV, respectively, are often used because of their strong promoter activity induced at the very-late phase of infection (~60 h post-infection (hpi)) [17,18]. Although the high level of *polh* expression requires many viral factors, including transactivators such as IE1 and very late factor 1 (VLF-1), and homologous region (*hr*) as an enhancer [19,20], the transient expression of several viral transactivators cannot achieve complete *polh* promoter activity as in infection [21]. Therefore, alteration of viral and cellular gene expression during baculovirus infection is also important for maximal recombinant protein production. In support of this notion, shutoff of the expressions of baculovirus late genes such as *vp39* and *gp64* has been observed [22,23]; for example, the expression level of *vp39* mRNA, encoding the capsid protein, was abundant at 24–36 hpi but significantly decreased at 48 hpi. Moreover, it has been reported that *polh* promoter activity is enhanced by the deletion of another very-late gene, *p10* [24]. Finally, the levels of almost all cellular mRNAs were reduced upon infection with AcMNPV by 24 hpi [25]. Collectively, these observations suggest that both the viral and host transcriptional machineries are hijacked to focus on the very-late gene expression, leading to a very high level of protein expression.

### 2.2. Genetic Modification of Baculovirus for Minimum Vector

The genetic modification of baculovirus by the reduction of its genome size is itself one of the strategies for the establishment of advanced BEVS. More than half of all baculovirus genes are predicted to be essential for viral propagation through gene expression, DNA replication and virion components [13]. Based on an extensive review of the literature, 94 of 156 genes of AcMNPV are predicted to be essential for cell culture propagation [26], while 55 of 141 genes of BmNPV are known to be essential for efficient propagation in cell culture by knockout analysis [27], suggesting that more than half of these genes are practically dispensable for infectious particle formation. Some reports have shown that several baculovirus genes exhibit negative effects on the expression of foreign genes in insect cells: *chiA* (chitinase), *v-cath* (cathepsin L-like proteinase) [28], *p10* [24] and *p74* (occlusion derived virus envelope protein) and *p26* (related to occlusion body formation) [29,30], and vector systems of the deletion mutants are already available using Oxford Expression Technologies such as flashBACULTRA^TM^. In addition, for multiple expression, several loci (*ctx*, *egt*, *39k*, *orf51*, *gp37*, *iap2*, and *odv-e56*) in the AcMNPV genome can be replaced with foreign gene expression cassettes [31]. However, baculovirus gene expression is not always simple and straightforward, and interactions among several genes have been reported (see also Section 2.1). Taka et al. reported evidence of such interactions; in their study, deletion of the BmNPV *orf11-12-13-14* cluster region (approximately 5 kbp) resulted in defective replication, although the viruses with the corresponding single knockouts were viable [32]. Therefore, to optimize BEVS with the minimum baculovirus genome, further studies on the baculovirus gene interaction are needed.

## 3. Baculovirus-Mediated Gene Delivery into Mammalian Cells

### 3.1. GP64-Mediated Entry into Mammalian Cells

Baculovirus has wide tropisms that permit its entry not only into the cells of insect hosts but also into various mammalian cells via its envelope glycoprotein, GP64 [33]. Although the critical receptors required for baculovirus entry into various cells have been unclear, several groups have provided insights into the host factors involved in the interactions with GP64. First, it has been reported that GP64 can bind to heparin sulfate proteoglycan (HSPG) on the cell surface [34] through the heparin binding motif of a 22 amino acid region between residues 271 and 292 of AcMNPV GP64 in a pH-dependent manner [35]. In particular, in contrast to other HSPG family members including SDC-2, SDC-4 and glypicans, Syndecan-1, which is mainly found in epithelial and plasma cells, was identified as a GP64 receptor in mammalian cells, but its expression level and binding affinity with baculovirus were not correlated [36]. In addition, we previously reported that GP64 can interact with cell surface phospholipids [6]. Entry of AcMNPV into mammalian cells has been inhibited by treatment with negatively charged lipids such as phosphatidic acid and phosphatidylinositol, and was reduced in mutant hamster cell lines deficient in phospholipid synthesis [6].

**Figure 1:**
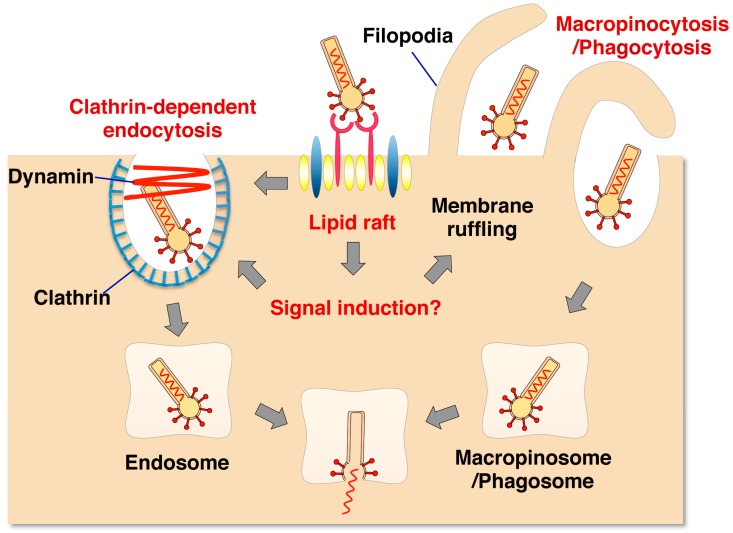
Putative model of internalization of baculovirus into mammalian cells. Baculovirus binds to HSPG or cellular receptor(s) present in the lipid raft. This association induces cellular remodeling through signal transduction. In clathrin-mediated endocytosis, baculovirus is internalized into the clathrin-coated pit. In macropinocytosis [37] or phagocytosis [40], filopodia formed by actin dynamics wrap the baculovirus into a macropinosome or phagosome. The viral genome is released from the endosome, macropinosome or phagosome through membrane fusion induced by low pH (Kataoka et al. [37], with modification).

After attachment and binding to cell surface molecules, baculovirus appears to be internalized by cells through lipid rafts [37]; this is supported by the observation that treatment with methyl-β-cyclodextrin, which removes cholesterol from cellular membranes, inhibits GP64-mediated internalization but not attachment to the cell surface [37]. On the other hand, the internalization pathway is still controversial, although it has been speculated to involve clathrin-mediated endocytosis [37,38,39] and/or macropinocytosis [37,39]. Laakkonen et al. reported that baculovirus is internalized by cells through dynamin-dependent phagocytosis but not through clathrin-mediated endocytosis or macropinocytosis, by the knockdown and expression of mutants of regulators of each pathway in HEK293 cells and HepG2 cells [40]. However, treatment of cells with inhibitors, gene knockdown or the expression of dominant-negative mutants for dynamin- and clathrin-mediated endocytosis has been shown to abrogate the internalization of AcMNPV into Huh7 cells, while inhibition of caveolin-mediated endocytosis had no effect [37]. In addition, inhibition of macropinocytosis reduced GP64-mediated internalization [37]. Collectively, these results indicate that cholesterol in the plasma membrane, dynamin- and clathrin-dependent endocytosis, and macropinocytosis play crucial roles in the entry of viruses bearing baculovirus GP64 into mammalian cells, but the internalization pathway may be dependent on cell type (Figure 1).

### 3.2. Modification of Envelope Proteins on Recombinant Baculovirus

Although baculovirus can enter mammalian cells, with especially efficient entry in hepatocytes [2], various modifications of the baculovirus envelope protein have been reported to increase transduction efficiency in a target cell type-dependent or -independent manner; an excess amount of GP64 [6], the recombinant baculovirus possessing an envelope glycoprotein derived from other viruses such as vesicular stomatitis virus (VSV) [41,42], rabies virus and mouse hepatitis virus [5], neuraminidase of influenza virus [43], and F protein of *Spodoptera exigua* multiple nucleopolyhedrovirus [44]. Recently, from a library of GP64 mutants generated by error-prone PCR, GP64 mutants with high transduction ability into human airway epithelia were obtained by serial passages of pseudotyped lentiviruses in primary human airway epithelia [45]. On the other hand, baculoviruses bearing heterologous proteins on viral particles have been shown to enhance the efficiency of gene delivery. For instance, baculoviruses bearing VP39, a major viral capsid protein, fused with the protein transduction domain of HIV TAT protein [46] and GP64 fused with the short peptide motif from gp350/220 of the Epstein-Barr virus for gene delivery to B-lymphocytic cells [47], the envelope protein of human endogenous retrovirus [48], single chain antibody fragments [49] and the Fc region of antibodies [50] have been reported. Notably, the display of Fc on the baculoviral particles allows a specific gene targeting the antigen-presenting cells expressing Fc receptors through phagocytosis. Among these strategies, display of the VSV G protein (VSVG) and heterologous peptide/protein via the GP64 anchor are the most widely adopted for enhancement of the in vitro and in vivo gene transduction efficiency of recombinant baculoviruses [5,6,51,52,53,54,55,56].

## 4. Immune Responses Induced by Baculovirus Infection in Mammalian Cells

### 4.1. Toll-Like Receptor-Dependent Pathway

**Figure 2:**
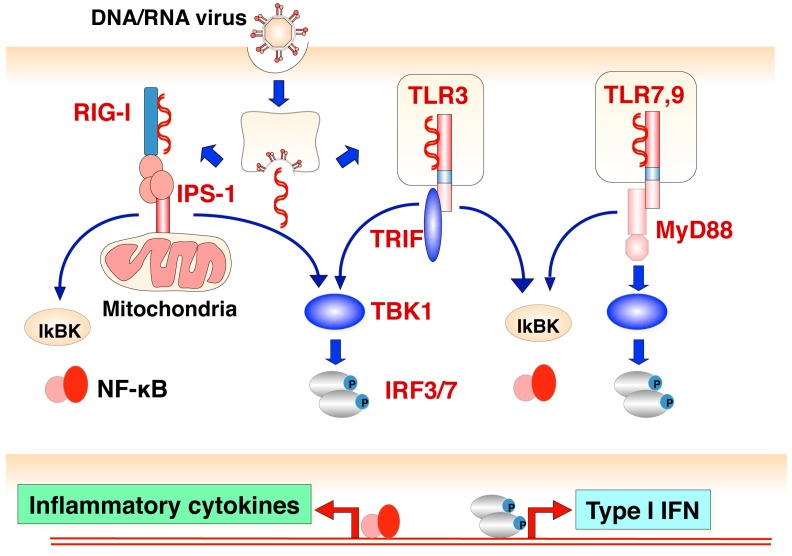
Induction of innate immune response by viral infection. Induction of IFN and pro-inflammatory cytokines is mainly initiated by the recognition of several viral components in mammalian cells. RIG-like receptors such as RIG-I and MDA5 (cytoplasmic sensors) recognize viral-specific double strand RNA, and then the IPS-1-mediated signaling pathway is activated. On the other hand, Toll-like receptors such as TLR9, TLR7 and TLR3 (endosomal sensors) recognize viral DNA, RNA and replicative intermediate double strand RNA, and then a TRIF- or Myd88-mediated signaling pathway is activated. Finally, these pathways trigger the activation of transcription factors such as NF-κB and IRF3 or IRF7, followed by the production of inflammatory cytokines and Type I IFNs, respectively.

AcMNPV was shown to possess a strong adjuvant activity to promote humoral and cellular immune responses by stimulation of interferons (IFNs) or pro-inflammatory cytokine production [8,10,11,57]. Because neither purified recombinant GP64 nor the inactivated baculovirus could induce antiviral immune responses [8,10,11,57], the responses may be caused by the internalized AcMNPV genome DNA, which contains a high level of unmethylated CpG DNA comparable to the genomes of *E. coli* and herpes simplex virus [9]. In general, virus infection induces activation of the TLR signaling pathway triggered by the recognition of viral genomes or transcripts (Figure 2) and an innate immune response against unmethylated CpG DNA is mediated by the TLR9/MyD88 signaling pathway [58,59]. In a study using knockout mice, we revealed that a TLR9/MyD88-dependent DNA recognition pathway participates in the production of type I IFNs, inflammatory cytokines and IFN-inducible chemokines and the activation of NF-κB in mouse immune cells such as macrophages or dendritic cells (DCs) in response to the AcMNPV genome [9,10]. Notably, the production of type I IFNs or inflammatory cytokines was more suppressed in IRF7-deficient peritoneal macrophages (PECs) or splenic CD11c^+^ DCs than in IRF3-deficient PECs or splenic CD11c^+^ DCs, while those antiviral responses were completely dependent on the TLR9/MyD88/IRF7 signaling pathway in plasmacytoid dendritic cells (pDCs). These observations suggest that the baculovirus-mediated innate immune response takes place in a cell-type-specific manner.

### 4.2. Toll-Like Receptor-Independent Pathway

**Figure 3:**
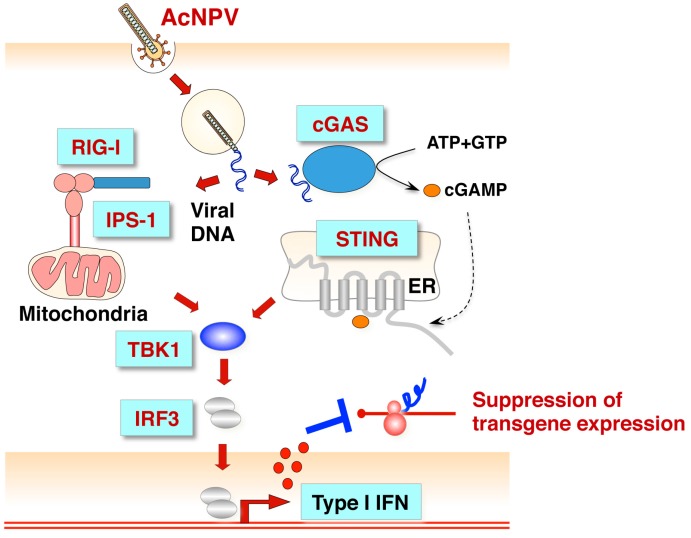
Innate immune response against baculovirus infection in mammalian non-immune cells. Baculovirus infection activates the RIG-I/IPS-1/TBK1/IRF3 or cGAS/STING/TBK1/IRF3 signaling pathway and induces Type I IFN production. Baculovirus DNA is recognized by cytoplasmic sensor RIG-I or cGAS [65], followed by activation of the IPS-1- or STING-mediated signaling pathway. The induction of Type I IFN production by IRF3 suppresses transgene expression by baculovirus [60].

On the other hand, with respect to baculovirus-mediated gene delivery into mammalian cells, the induction of an antiviral immune response suppresses an efficient transgene expression [60]. Previously, we examined the effects of the innate immune responses on gene expression by recombinant baculoviruses in mouse embryonic fibroblasts (MEFs) [60]. Although the innate immune response against unmethylated CpG DNA was mediated by the TLR9/MyD88 signaling pathway in macrophages or DCs [58,59], the production of type I IFN and inflammatory cytokines in MyD88-deficient MEFs infected with the recombinant baculovirus was not significantly different from that of the wild type, resulting in the suppression of reporter gene expression. On the other hand, reporter gene expression was enhanced in accordance with the suppression of IFN-β production in stimulator of IFN genes (STING)-, TANK binding kinase 1 (TBK1)-, IFN-β promoter stimulator 1 (IPS-1) or IRF3-deficient MEFs, but not in those deficient for Z-DNA binding protein 1 (ZBP1)/DAI or IRF7. A cyclic GMP-AMP (cGAMP) synthase (cGAS) has been reported as a candidate for an upstream cytosolic DNA sensor of STING [61,62]. In the cGAS-STING signaling axis, cGAMP produced by cGAS acts as a second messenger in cells stimulated by cytosolic DNAs, activates STING, and is involved in the antiviral response against DNA viruses [63,64]. Recently, it has been reported that RAB2B-GARIL5 complex, which participate in activation of the cGAS-STING signaling pathway, inhibits the expressions of IFN-β and CXCL10 induced by baculovirus and by a modified vaccinia virus Ankara strain (MVA) [65]. These results suggest that the cGAS/STING/TBK1/IRF3 and IPS-1/TBK1/IRF3 signaling axes contribute to the antiviral response in mammalian non-immune cells upon infection with recombinant AcMNPV (Figure 3).

## 5. Baculovirus for Gene Therapy

Based on its ability to deliver transgenes into various mammalian cells, baculovirus has been applied to cancer therapy for effective suppression of tumor development. Baculovirus has also been utilized as a toxin vector expressing diphtheria toxin A to eliminate malignant glioma cells in the brain [66] or a toxin vector expressing herpes simplex virus thymidine kinase (HSVtk), which mediates cell death triggered in the presence of ganciclovir to eliminate glioblastoma [67]. HSVtk was expressed specifically in glioblastoma under the control of an HMBG2 promoter with low activity in normal cells [67]. In addition, baculovirus-mediated expression of a tumor suppressor gene, normal epithelial cell specific-1 (NES1) has been reported to inhibit growth of gastric cancer cells [68].

**Figure 4:**
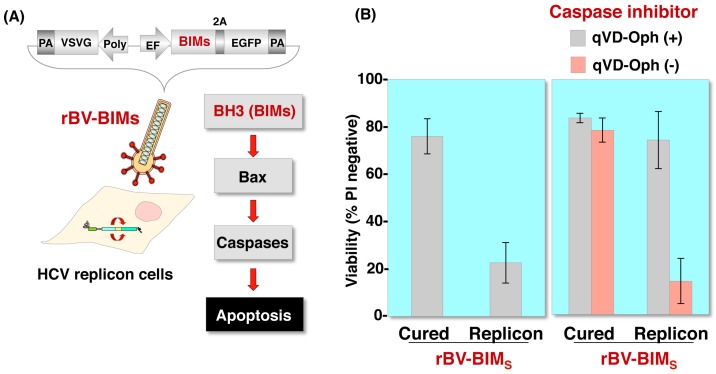
Induction of apoptosis in cells replicating HCV RNA by a recombinant baculovirus expressing a proapoptotic protein. (**A**) Structure of a recombinant baculovirus, rBV-BIM_S_, carrying cDNAs of BIMs 2A peptides, and enhanced green fluorescent protein (EGFP) under the control of the elongation factor 1α promoter. The apoptosis pathway was induced by BIMs. (**B**) HCV replicon cells (HCV-RNA-replicating Huh7 cells) and cured cells (replicon cells from which HCV-RNA was eliminated) were infected with rBV-BIM_S_ at an MOI of 500, and cell viability was determined at 24 h post infection. rBV-BIM_S_ can induce apoptosis more efficiently against HCV replicon cells than cured cells (left). HCV replicon cells and cured cells were infected with rBV-BIM_S_ at an MOI of 500 in the presence or absence of 20 μM qVD-Oph (caspase inhibitor), and cell viability was determined at 24 h post infection. The results show that infection with rBV-BIM_S_ selectively induced apoptosis in HCV replicon cells through the activation of Bcl-2 family proteins (right).

Moreover, wild type baculovirus has a strong adjuvant activity that can protect mice from lethal virus infections such as influenza or encephalomyocarditis virus [8,11]. Wild type AcMNPV-induced antitumor acquired immunity, which can involve activities such as the activation of tumor-specific cytotoxic lymphocytes (CTLs), NK cells or production of antibodies, can suppress the tumor growth of mouse melanoma [12]. Our previous observations also suggest possible baculovirus applications for gene therapy. Because baculovirus induces antiviral innate immune responses via the IPS-1-TBK1-IRF3 pathway [60], we focused on the enhancement of transgene expression in immune-deficient cells. Upon infection with hepatitis C virus (HCV), innate immunity was impaired by the cleavage of IPS-1 by the viral protease NS3/4A [69], leading to the enhancement of gene expression by the recombinant baculovirus. Further, infection with the recombinant baculovirus expressing the BH3-only protein, BIM_S_, a potent inducer of apoptosis [70], resulted in selective cell death in HCV-positive cells [60] (Figure 4). These observations indicate that this characteristic of baculovirus might be useful for selective gene transduction in cells with impaired innate immunity arising from infection with various viruses such as HIV or viruses with an antagonist targeting a host immune response.

## 6. Conclusions and Future Perspectives

The continued development of baculovirus technology contributes to numerous advances in basic biology, especially in structural biology, biochemistry and cell biology. Baculoviruses have also been developed as safe and efficient gene delivery vectors, based on their broad entry tropism and replication-deficiency in mammalian cells. However, anti-baculoviral responses are induced by the recognition of unmethylated CpG DNA of the baculovirus genome in mammalian cells in a cell-type dependent manner, meditated by the TLR9/MyD88/IRF7 pathway in immune cells and the cGAS/STING/TBK1/IRF3 or IPS-1/TBK1/IRF3 pathways in non-immune cells such as fibroblasts, resulting in inefficient expression of the transgenes delivered into mammalian cells. On the other hand, these responses can also be exploited for more effective vaccination, since the induction of a strong adjuvant activity against immune cells is valuable in this context. Therefore, the combination/contamination of live baculovirus in BEVS-derived purified recombinant proteins or VLPs can provide benefits for inoculated patients. Recently, Heinimäki et al. reported that, compared to highly purified VLPs (i.e., VLPs purified by anion exchange chromatography), crudely purified VLPs (VLPs purified by sucrose gradient ultracentrifugation) from recombinant baculovirus-infected insect cells, which contained live baculovirus, promoted immunogenicity against B and T cells [71], while this adjuvant effect was lost by the inactivation of baculovirus. However, for safety concerns, the BEVS-derived recombinant proteins or VLPs that have been approved and are commercially available as vaccines are always purified, because baculovirus gene expression in humans and its potential side effects are still controversial. Although the cellular gene expression profiles in mammalian cells do not change significantly upon infection with baculovirus [72], several reports have shown that the potent baculovirus transactivator IE2 can enhance not only the expression of baculovirus genes [73] but also the mammalian promoter activity in mammalian cells [74,75]. Moreover, the generation of recombinant baculovirus and the use of the commercially available recombinant proteins produced by BEVS are regulated in accordance with the terms of The Cartagena Protocol on Biosafety to the Convention on Biological Diversity, which is an international agreement among 170 signatory countries, including the EU, China and Japan, to ensure that adequate safety is maintained when living modified organisms are handled or transported. To overcome the limitations for practical use of BEVS-derived vaccines containing live baculovirus, further technical development is needed. Because the induction of innate immune response by live baculovirus is mediated by infection event and resulting internalization of genome DNA into cells, the establishment of deletion mutant baculovirus of both genes essential for the virus infectivity (e.g., viral glycoprotein gene *gp64*) and viral gene expression (e.g., viral essential/potent transactivator genes *ie1* or *ie2*) can be useful for the next-generation BEVS with the enhancement of safety. However, the expression of those genes in trans to recover infectious virus production sometimes induces the emergence of revertant virus [54]. In our previous study, during amplification of a *gp64*-null pseudotype baculovirus in *gp64*-expressing insect cells, we observed the high-frequency appearance of a replication-competent virus incorporating the *gp64* gene into the viral genome, while the expression of VSVG instead of GP64 could avoid the emergence of revertants [54]. These reports highlight numerous concerns regarding the future use of BEVS in the development of revolutionary vaccines, including recombinant proteins and live baculoviruses as antigens and natural adjuvants, respectively. Therefore, additional investigations on the possible side effects of the infection of mammalian cells with baculovirus are required for the safety assessment, with a focus on the alteration of cellular gene expressions or the induction of signaling pathways.
